# The Prognostic Value of Circulating Tumor DNA for Clinical Outcomes in Patients Undergoing Hematopoietic Cell Transplantation: A Systematic Review and Meta-Analysis

**DOI:** 10.3390/ijms27115076

**Published:** 2026-06-04

**Authors:** Do Tung Dac, Hirokazu Tanaka, Akiyoshi Takami, Jorge Luis Espinoza

**Affiliations:** 1 Faculty of Health Sciences, Kanazawa University, Kanazawa 9200942, Ishikawa, Japan; dotungdac@stu.kanazawa-u.ac.jp; 2 Department of Hematology, Faculty of Medicine, Kindai University, Osaka-Sayama 5898511, Osaka, Japan; htanaka@med.kindai.ac.jp; 3 Department of Hematology, Faculty of Medicine, Aichi Medical University, Nagakute 4801195, Aichi, Japan; takami-knz@umin.ac.jp

**Keywords:** ctDNA, minimal residual disease, MRD, hematopoietic cell transplantation, relapse, survival, liquid biopsy, meta-analysis

## Abstract

Relapse remains the leading cause of treatment failure following hematopoietic cell transplantation (HCT) for hematologic malignancies. Circulating tumor DNA (ctDNA) has emerged as a promising minimally invasive biomarker for measurable residual disease (MRD) assessment and early relapse detection; however, the prognostic significance of ctDNA in the post-transplant setting has not been comprehensively synthesized. We conducted a systematic review and meta-analysis in accordance with PRISMA guidelines and registered the protocol in PROSPERO (CRD420261392100). PubMed, Embase, Web of Science, EBSCO, Cochrane CENTRAL, and supplementary sources were searched through November 2025. Eligible studies evaluated tumor-specific ctDNA or tumor-informed/tumor-associated cfDNA in patients undergoing allogeneic or autologous HCT for hematologic malignancies. Random-effects meta-analyses were performed for relapse/progression, overall survival (OS), and relapse-free/progression-free survival (RFS/PFS). Studies evaluating total cfDNA quantity, methylation-based cfDNA profiling, cfRNA, or chimerism-only monitoring were synthesized narratively. Ten observational cohort studies comprising 883 patients met inclusion criteria. Across acute leukemias, lymphomas, multiple myeloma, and myelodysplastic syndromes, ctDNA/cfDNA positivity was consistently associated with adverse outcomes. The pooled hazard ratio (HR) for relapse or disease progression was 12.57 (95% CI: 4.59–34.46; *p* < 0.001), while pooled HRs were 7.45 (95% CI: 4.11–13.48; *p* < 0.001) for OS and 4.46 (95% CI: 2.22–8.97; *p* < 0.001) for RFS/PFS. Although statistical heterogeneity was low, interpretation was limited by the relatively small number of studies contributing to each pooled endpoint. Narrative evidence additionally suggested that broader circulating nucleic acid approaches may provide complementary information regarding graft-versus-host disease, infection, and other post-transplant complications. Tumor-specific ctDNA positivity is consistently associated with increased relapse risk and inferior survival outcomes following HCT. These findings support further investigation of ctDNA-based MRD monitoring as a promising non-invasive biomarker for post-transplant molecular surveillance and risk stratification. However, prospective multicenter validation studies, assay standardization, and ctDNA-guided interventional trials remain necessary before routine clinical implementation can be recommended.

## 1. Introduction

Hematopoietic cell transplantation (HCT) remains a cornerstone of curative therapy for a broad spectrum of hematologic malignancies, including acute myeloid leukemia (AML), acute lymphoblastic leukemia (ALL), lymphomas, multiple myeloma (MM), and myelodysplastic syndromes (MDS) [1]. Over recent decades, advances in conditioning regimens, donor selection, graft engineering, infection prevention, and supportive care have substantially improved transplant outcomes and reduced non-relapse mortality (NRM). Nevertheless, disease relapse remains the leading cause of treatment failure after HCT, affecting approximately 30–50% of patients depending on disease subtype, disease status at transplantation, conditioning intensity, and transplant modality [2,3,4]. Once overt relapse occurs following HCT, prognosis is generally poor, underscoring the importance of sensitive post-transplant monitoring strategies capable of identifying residual disease before clinical recurrence develops.

Conventional measurable residual disease (MRD) monitoring methods, including multiparameter flow cytometry (MFC) and allele-specific polymerase chain reaction (PCR)-based assays, have improved the detection of residual malignant cells and refined post-transplant risk stratification. However, these approaches have important limitations. Bone marrow-based monitoring is invasive and susceptible to sampling bias, particularly in diseases characterized by spatial heterogeneity or patchy marrow involvement. In addition, conventional assays may fail to fully capture clonal evolution and emerging resistant subclones during post-transplant disease progression [5,6]. Variability in assay platforms, sensitivity thresholds, and interpretation criteria across centers also limits standardization and broader clinical applicability. These limitations have stimulated increasing interest in minimally invasive liquid biopsy approaches for post-HCT surveillance.

Circulating tumor DNA (ctDNA), which consists of tumor-derived DNA fragments released into the bloodstream through apoptosis, necrosis, or active secretion by malignant cells, has emerged as a promising biomarker for real-time molecular disease monitoring [7]. Tumor-specific ctDNA assays may detect molecular relapse weeks to months before overt hematologic or radiographic recurrence becomes clinically evident [8,9]. Unlike conventional single-site tissue or marrow sampling, ctDNA potentially provides a more comprehensive representation of tumor heterogeneity and evolving clonal architecture [10]. Serial ctDNA monitoring has therefore generated substantial interest as a tool for dynamic MRD assessment, relapse prediction, and longitudinal disease surveillance across multiple hematologic malignancies.

Importantly, circulating nucleic acid approaches in the HCT setting are biologically heterogeneous and should not be considered interchangeable methodologies. Tumor-specific ctDNA refers to circulating tumor-derived nucleic acids identified through mutation-based, rearrangement-based, or patient-informed assays. In contrast, total cell-free DNA (cfDNA) quantification reflects overall circulating DNA burden without tumor specificity and may be influenced by tissue injury, inflammation, endothelial damage, infection, or conditioning-related toxicity. Methylation-based or whole-genome bisulfite sequencing (WGBS) approaches evaluate genome-wide cfDNA signatures that may reflect tissue injury and immune activation rather than direct molecular relapse [11,12]. Similarly, cell-free RNA (cfRNA) profiling may provide insights into inflammatory and immune-related processes, whereas chimerism assays assess donor–recipient hematopoietic composition rather than tumor-derived DNA directly [13]. Distinguishing these methodologies is essential when interpreting the growing liquid biopsy literature in HCT populations.

In the post-transplant setting, ctDNA-guided monitoring may ultimately support risk-adapted management strategies through earlier identification of impending relapse [14,15]. Potential interventions triggered by molecular relapse detection could include tapering of immunosuppression, donor lymphocyte infusion, targeted therapies, bispecific antibodies, or cellular immunotherapies administered at a lower disease burden [16]. However, despite increasing interest in ctDNA for post-HCT surveillance, the available literature remains fragmented. Published studies vary substantially in underlying diseases, transplant modalities, assay platforms, ctDNA targets, timing of sample collection, and endpoint definitions [17,18]. Moreover, prior reports have frequently combined biologically distinct liquid biopsy methodologies—including total cfDNA, methylation-based profiling, and chimerism monitoring—within the same analytical framework, complicating interpretation of the prognostic significance of tumor-specific ctDNA.

To address these gaps, we conducted a systematic review and meta-analysis evaluating the prognostic significance of tumor-specific ctDNA and tumor-informed cfDNA monitoring in patients undergoing HCT for hematologic malignancies. We specifically aimed to quantify associations between ctDNA positivity and relapse, overall survival (OS), and relapse-free/progression-free survival (RFS/PFS), while separately synthesizing non-tumor-specific circulating nucleic acid approaches narratively because of their biological and methodological heterogeneity. Through this approach, we sought to provide a clearer assessment of the current evidence supporting ctDNA-based MRD monitoring in the transplant setting and to identify key priorities for future clinical translation and research.

## 2. Materials and Methods

### 2.1. Study Design and Reporting Standards

This systematic review and meta-analysis were conducted in accordance with the Preferred Reporting Items for Systematic Reviews and Meta-Analyses (PRISMA 2020) guidelines [19]. The review protocol was prospectively registered in the International Prospective Register of Systematic Reviews (PROSPERO; CRD420261392100).

Eligible studies were evaluated according to the PICO framework as follows:

- Population: Patients of any age with hematologic malignancies undergoing allogeneic or autologous HCT, including AML, ALL, malignant lymphoma, MDS, MM, and related disorders.
- Index prognostic factor: Detection of tumor-specific ctDNA or tumor-informed/tumor-associated cfDNA for MRD assessment at any time point before or after HCT.
- Comparator: Patients with undetectable ctDNA/cfDNA at the same assessment time point.
- Outcomes: Primary outcomes included relapse, cumulative incidence of relapse (CIR), RFS, and PFS. Secondary outcomes included OS, graft-versus-host disease (GVHD), graft failure, transplant-related mortality (TRM), and other post-transplant complications.
- Study design: Prospective or retrospective observational cohort studies reporting hazard ratios (HRs) with 95% confidence intervals (CIs), or providing sufficient data for HR reconstruction. Reviews, editorials, conference abstracts without sufficient data, case reports, and non-English studies were excluded.

### 2.2. Information Sources and Search Strategy

A comprehensive literature search was conducted from database inception through 30 November 2025, using the following electronic databases:

- PubMed/MEDLINE;
- Embase;
- Web of Science;
- EBSCO;
- Cochrane Central Register of Controlled Trials (CENTRAL).

Additional supplementary searches were performed using Google Scholar and manual screening of reference lists from relevant articles and reviews. Clinical trial registries, including ClinicalTrials.gov and the WHO International Clinical Trials Registry Platform (ICTRP), were also searched for ongoing or unpublished studies.

The search strategy combined controlled vocabulary (MeSH and Emtree terms) with free-text keywords related to:

1. Circulating tumor DNA and liquid biopsy;
2. Measurable residual disease;
3. Hematopoietic cell transplantation;
4. Relapse and survival outcomes.

Representative search terms included: (“circulating tumor DNA” OR ctDNA OR “cell-free DNA” OR cfDNA OR “liquid biopsy” OR “minimal residual disease” OR MRD) AND (“hematopoietic cell transplantation” OR HSCT OR allo-HCT OR ASCT OR “bone marrow transplantation”) AND (relapse OR survival OR prognosis).

Search strategies were adapted appropriately for individual databases.

### 2.3. Study Selection and Data Extraction

Two independent reviewers (D.T.D. and J.L.E.) screened titles and abstracts according to predefined eligibility criteria. Potentially relevant articles subsequently underwent full-text review. Disagreements regarding study eligibility were resolved through discussion and consensus or adjudication by a third reviewer (A.T.). Data extraction was performed using a standardized pilot-tested form. Extracted variables included:

- Study characteristics (author, year, country, design);
- Patient demographics and disease subtype;
- Transplant characteristics;
- ctDNA/cfDNA assay methodology;
- Sampling time points;
- Outcome measures;
- HRs and corresponding 95% CIs.

When multiple time points were reported, clinically relevant landmark assessments (e.g., pre-HCT, day +30, day +90/+100 post-HCT) were preferentially extracted.

When available, multivariable-adjusted HRs were preferentially extracted over univariable estimates. If HRs were not directly reported, they were reconstructed from Kaplan–Meier curves using the method described by Tierney et al. [13].

**Figure 1:**
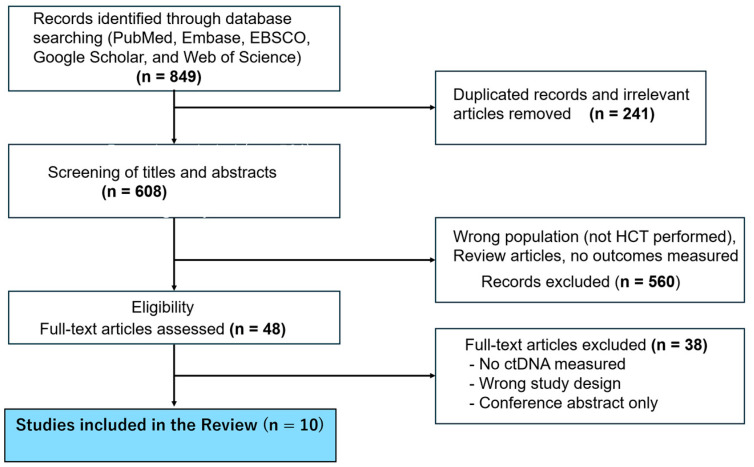
PRISMA flow diagram: The systematic search of PubMed, Embase, Web of Science, Google Scholar, and EBSCO, along with manual screening, yielded 849 records. Following the removal of 241 duplicates, 608 records were screened by title/abstract. Full-text assessment of 48 articles against eligibility criteria led to the final inclusion of 10 observational cohort studies in the systematic review and meta-analysis.

Our systematic search identified 849 records across electronic databases and supplementary searches. After removal of 241 duplicates, 608 unique studies underwent title and abstract screening. Forty-eight full-text articles were assessed for eligibility, of which 10 observational cohort studies (6 retrospective and 4 prospective) met inclusion criteria for the final systematic review (Figure 1). Studies eligible for quantitative meta-analysis were further restricted to those reporting extractable HRs for tumor-specific ctDNA/cfDNA outcomes.

### 2.4. Risk of Bias Assessment

Risk of bias was independently assessed by two reviewers using the **Quality In Prognosis Studies (QUIPS)** tool [19]. This instrument evaluates six domains:

1. Study participation;
2. Study attrition;
3. Prognostic factor measurement;
4. Outcome measurement;
5. Study confounding;
6. Statistical analysis and reporting.

Each domain was classified as low, moderate, or high risk of bias. Discrepancies were resolved through consensus discussion.

### 2.5. Data Synthesis and Statistical Analysis

All statistical analyses were performed using R software (version 4.3.2) with the meta and metafor packages.

Only studies evaluating tumor-specific ctDNA or tumor-informed/tumor-associated cfDNA MRD assays with extractable HRs for relapse, OS, RFS, or PFS were eligible for quantitative synthesis. Studies evaluating total cfDNA quantity, methylation-based cfDNA profiling, cfRNA, or chimerism-only monitoring were synthesized narratively because these assays do not directly measure tumor-specific ctDNA and demonstrated substantial biological and methodological heterogeneity.

Pooled HRs and corresponding 95% CIs were calculated using a generic inverse-variance random-effects model (DerSimonian and Laird method) to account for anticipated heterogeneity in disease populations, transplant modalities, assay methodologies, and sampling schedules.

Statistical heterogeneity was assessed using Cochran’s Q test and the I^2^ statistic. An I^2^ value > 50% was considered indicative of substantial heterogeneity. Because relatively few studies contributed to several pooled analyses, pooled estimates and heterogeneity statistics were interpreted cautiously.

Pre-specified subgroup analyses according to disease subtype, HCT modality, timing of ctDNA assessment, and assay platform were planned. However, formal subgroup analyses were limited by the small number of eligible studies contributing to each pooled endpoint. Sensitivity analyses were planned by sequential exclusion of studies judged to have high overall risk of bias.

Publication bias was evaluated visually using funnel plots and statistically using Egger’s regression test when sufficient studies were available for pooled analysis. Formal publication bias assessment was limited by the small number of studies contributing to several pooled outcomes.

## 3. Results

### 3.1. Characteristics of Included Studies

The 10 included studies encompassed a total of 883 patients, with sample sizes ranging from 10 to 177 participants per study. Studies were conducted internationally, with most originating from the United States (n = 5) [20,21,22,23,24]. Additional studies were conducted in Japan [25], Denmark [26], Turkey [27], South Korea [28], and Germany [29]. The spectrum of underlying hematologic malignancies was broad and included AML, ALL, MDS, MM, and various malignant lymphomas. Overall, six studies employed retrospective observational cohort designs [20,21,22,25,26,27], while four were prospective cohort or pilot studies [23,24,28,29]. Eight studies focused on allo-HCT [21,22,23,24,25,26,27,29], whereas two evaluated autologous HCT populations [20,28].

The methodologies used for circulating nucleic acid assessment were heterogeneous and included targeted next-generation sequencing (NGS), droplet digital PCR (ddPCR), WGBS, spectrophotometric total cfDNA quantification, and real-time quantitative PCR (RQ-PCR) chimerism assays. Because these approaches capture biologically distinct processes, studies were categorized into: (1) tumor-specific ctDNA studies, (2) tumor-associated cfDNA/cfDNA-MRD studies, and (3) non-tumor-specific approaches, including total cfDNA quantification, methylation-based cfDNA profiling, and chimerism-based monitoring. The main characteristics of included studies are summarized in Table 1.

### 3.2. Survival Outcomes

Eight studies evaluated associations between circulating nucleic acid biomarkers and OS, RFS, or PFS. Because included studies assessed biologically distinct biomarker approaches, pooled quantitative analyses were restricted to tumor-specific ctDNA/cfDNA studies with extractable hazard ratios, whereas total cfDNA, methylation-based profiling, and chimerism studies were interpreted qualitatively.

Among tumor-specific ctDNA studies, Dhakal et al. (2022) demonstrated that ctDNA positivity at 3 months following autologous HCT in MM independently predicted inferior PFS (HR = 5.6; 95% CI: 1.8–17.0; *p* = 0.0003) [20]. Similarly, Nakamura et al. (2019) reported that ctDNA positivity at 3 months post-allogeneic HCT was strongly associated with inferior OS in AML/MDS (HR = 8.04; 95% CI: 1.66–38.88; *p* < 0.01) [19]. Herrera et al. (2016) found that post-transplant ctDNA detection in lymphoma patients was associated with significantly inferior PFS (HR = 3.9; 95% CI: 1.6–9.5; *p* = 0.003) [15].

Among tumor-associated cfDNA studies, Pasca et al. (2023) demonstrated that tumor-informed cfDNA-MRD positivity at day +90 after allo-HCT in myeloid malignancies was strongly associated with inferior RFS and OS, with a 2-year cumulative incidence of relapse of 76% among cfDNA-positive patients compared with 21% among negative patients (*p* < 0.001) [24]. Patel et al. (2025) likewise reported that tumor-associated cfDNA positivity was associated with increased mortality and relapse risk following allogeneic HCT in AML (HR for OS = 5.4; 95% CI: 2.5–11.8; *p* < 0.0001) [14].

Waterhouse et al. (2022) demonstrated that longitudinal patient-specific cfDNA monitoring enabled early relapse detection, including extramedullary relapse, and was associated with inferior survival outcomes [29]. In contrast, Yegin et al. (2020), who evaluated total cfDNA quantity rather than tumor-specific ctDNA, did not identify a statistically significant association between cfDNA levels and OS [27].

Across studies eligible for quantitative synthesis, pooled meta-analysis demonstrated that ctDNA/cfDNA positivity was significantly associated with inferior OS (pooled HR = 7.45; 95% CI: 4.11–13.48) and inferior RFS/PFS (pooled HR = 4.46; 95% CI: 2.22–8.97). Although statistical heterogeneity was low, interpretation was limited by the relatively small number of studies contributing to each pooled endpoint. A summary of survival outcome data is presented in Table 2.

### 3.3. Prediction of Graft-Versus-Host Disease and Other Complications

Beyond relapse surveillance, emerging evidence suggests that broader cell-free nucleic acid profiling approaches may also provide clinically relevant information regarding non-relapse complications following HCT. Cheng et al. (2022) utilized low-coverage whole-genome bisulfite sequencing of plasma cfDNA to simultaneously evaluate multiple post-transplant complications, including GVHD, infection, relapse, and graft failure [23]. Their findings demonstrated that methylation-based cfDNA profiling could capture tissue injury and immune-related signals associated with post-HCT complications, supporting the potential role of broader liquid biopsy approaches beyond tumor-specific relapse monitoring.

### 3.4. Engraftment and Transplant-Related Mortality

Several studies additionally explored associations between circulating nucleic acid biomarkers and transplant-related complications, including TRM, NRM, and graft-related outcomes. Yegin et al. (2020) reported an inverse relationship between pre-transplant total cfDNA levels and transplant-related complications, with lower total cfDNA levels associated with increased complications and mortality [27]. Because this study measured total cfDNA quantity by spectrophotometry rather than tumor-specific ctDNA, the biological interpretation of these findings likely differs from mutation-informed MRD approaches.

Haugaard et al. (2019) demonstrated that highly sensitive RQ-PCR chimerism monitoring predicted relapse approximately seven months before morphological recurrence in pediatric ALL/AML patients, while also showing associations with transplant-related outcomes [26]. Similarly, Herrera et al. (2016) reported associations between post-transplant ctDNA positivity and increased non-relapse mortality among lymphoma patients undergoing allo-HCT [21] (Table 3).

### 3.5. Relapse Prediction

All included studies evaluated relapse-related outcomes, although substantial heterogeneity existed in biomarker methodology and study design (Table 4). To minimize biological heterogeneity, quantitative pooling was restricted to tumor-specific ctDNA/cfDNA studies with extractable hazard ratios, while total cfDNA, methylation-based profiling, and chimerism studies were interpreted qualitatively.

Among tumor-specific ctDNA studies, Dhakal et al. (2022) reported that ctDNA positivity at 3 months post-autologous HCT strongly predicted relapse in multiple myeloma and outperformed multiparameter flow cytometry MRD assessment [20]. Nakamura et al. (2019) demonstrated that serial ctDNA measurements at 1 and 3 months after allo-HCT were highly predictive of relapse in AML/MDS, with increasing ctDNA kinetics providing particularly high sensitivity for impending recurrence [25]. Herrera et al. (2016) found that post-transplant ctDNA detection was associated with significantly increased disease progression risk in lymphoma patients [21]. Kim et al. (2024) further demonstrated that ctDNA monitoring identified high-risk lymphoma patients who may benefit from intensified upfront transplant strategies [28].

Among tumor-associated cfDNA studies, Pasca et al. (2023) showed that day +90 cfDNA-MRD positivity was associated with a markedly increased cumulative incidence of relapse in myeloid malignancies [24]. Patel et al. (2025) similarly reported strong associations between tumor-associated cfDNA positivity and relapse following allogeneic HCT in AML [22]. Waterhouse et al. (2022) demonstrated that longitudinal patient-specific cfDNA monitoring enabled early detection of relapse, including extramedullary disease [29].

In contrast, Yegin et al. (2020) evaluated total cfDNA concentration rather than tumor-specific ctDNA and reported that lower total cfDNA levels were associated with increased relapse risk in AML [27]. Cheng et al. (2022), whose primary focus was post-transplant complication profiling rather than MRD surveillance, reported that methylation-based cfDNA profiling could also capture relapse-associated signals [23]. Haugaard et al. (2019) demonstrated that chimerism-based MRD monitoring using highly sensitive RQ-PCR predicted relapse approximately seven months before morphological recurrence in pediatric leukemia [26].

Across studies eligible for quantitative synthesis, the pooled HR for relapse or disease progression associated with ctDNA/cfDNA positivity was 12.57 (95% CI: 4.59–34.46), supporting a strong association between molecular positivity and adverse post-transplant oncologic outcomes.

### 3.6. Risk of Bias Assessment

Risk of bias assessment using the QUIPS tool demonstrated an overall moderate risk of bias across included studies. Most studies showed low risk of bias in outcome measurement and statistical reporting domains, reflecting the use of objective clinical endpoints and generally transparent statistical analyses.

The most frequent sources of potential bias involved the prognostic factor measurement and study confounding domains. Variability in ctDNA assay methodologies, timing of sample collection, ctDNA thresholds, and plasma processing protocols contributed to moderate methodological heterogeneity across studies. In addition, several retrospective studies lacked comprehensive multivariable adjustment for established prognostic factors, including disease risk, cytogenetic abnormalities, conditioning intensity, donor type, or GVHD status.

Studies evaluating total cfDNA quantity or non-tumor-specific approaches were generally judged to have a higher risk of bias due to limitations in biological specificity and assay standardization. Retrospective studies additionally demonstrated increased susceptibility to attrition and participation bias compared with prospective cohort studies. A detailed summary of the QUIPS assessment is presented in Table 5.

Risk of Bias Domains (QUIPS):

1. Study Participation;
2. Study Attrition;
3. Prognostic Factor Measurement;
4. Outcome Measurement;
5. Study Confounding;
6. Statistical Analysis and Reporting.

Interpretation: Most studies demonstrated low risk of bias in outcome measurement and statistical reporting domains. Moderate or high-risk judgments were primarily driven by variability in ctDNA assay methodologies, inconsistent sampling schedules, retrospective study design, incomplete adjustment for established prognostic variables, and limited sample sizes.

### 3.7. Meta-Analysis of ctDNA Positivity and Survival Outcomes

Only studies evaluating tumor-specific ctDNA or tumor-informed/tumor-associated cfDNA with extractable hazard ratios were included in the quantitative meta-analysis. Studies evaluating total cfDNA quantity, methylation-based cfDNA profiling, cfRNA, or chimerism-only monitoring were excluded from pooled analyses because of substantial biological and methodological heterogeneity.

The summarized HR data used in the pooled analyses are presented in Table 6. Meta-analysis demonstrated statistically significant associations between ctDNA/cfDNA positivity and adverse clinical outcomes across all primary endpoints.

**Figure 2:**
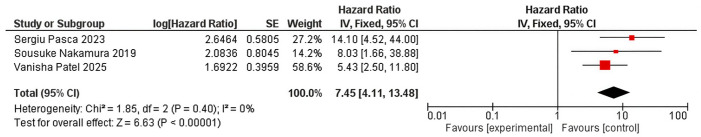
Forest Plot for the Association Between ctDNA/cfDNA Positivity and Overall Survival (OS) [22,24,25]. The squares represent individual study HRs; a diamond represents the pooled HR from the random-effects meta-analysis. Horizontal lines denote 95% confidence intervals. The vertical dashed line indicates the null effect (HR = 1). I^2^ quantifies statistical heterogeneity.

**Figure 3:**
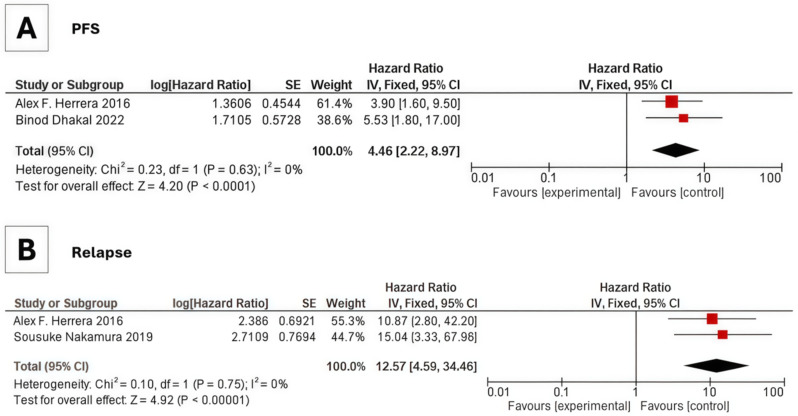
Forest Plot for the Association Between ctDNA Positivity and Progression-Free Survival and Relapse. Forest plot of the pooled hazard ratio (HR) for the association between ctDNA positivity and Progression-Free Survival reported by all eligible studies [20,21] (**A**), and Forest plot of the pooled hazard ratio (HR) for the association between ctDNA positivity and Relapse [21,25] (**B**). In (**A**,**B**), squares represent individual study HRs; a diamond represents the pooled HR from the random-effects meta-analysis. Horizontal lines denote 95% confidence intervals. The vertical dashed line indicates the null effect (HR = 1). I^2^ quantifies statistical heterogeneity.

For overall survival, ctDNA/cfDNA positivity was associated with significantly inferior OS (pooled HR = 7.45; 95% CI: 4.11–13.48; *p* < 0.001) (Figure 2). Similarly, pooled analysis demonstrated significantly inferior RFS/PFS among ctDNA/cfDNA-positive patients (pooled HR = 4.46; 95% CI: 2.22–8.97; *p* < 0.001) (Figure 3A). Finally, ctDNA/cfDNA positivity was strongly associated with relapse or disease progression (pooled HR = 12.57; 95% CI: 4.59–34.46; *p* < 0.001) (Figure 3B).

Although statistical heterogeneity across pooled analyses was low, interpretation should remain cautious because only a limited number of studies contributed to each endpoint, thereby reducing the power of heterogeneity statistics to detect true between-study variability.

## 4. Discussion

This systematic review and meta-analysis demonstrate that tumor-specific ctDNA positivity is consistently associated with increased relapse risk and inferior survival outcomes following HCT across multiple hematologic malignancies. Quantitative synthesis revealed statistically significant associations between ctDNA/cfDNA positivity and relapse or disease progression, OS, and RFS/PFS. Collectively, these findings support the potential role of ctDNA-based MRD monitoring as a promising non-invasive biomarker for post-transplant molecular surveillance and risk stratification.

Our findings are consistent with prior studies supporting the clinical utility of ctDNA-based MRD assessment in hematologic malignancies and extend these observations specifically to the HCT setting [17,30]. One of the most clinically relevant features of ctDNA monitoring is the ability to detect molecular relapse weeks to months before overt hematologic or radiographic recurrence becomes clinically apparent. Several included studies demonstrated that serial ctDNA kinetics may provide particularly informative prognostic signals, with rising ctDNA levels preceding clinical relapse by substantial intervals [31]. This capability is especially relevant after allo-HCT, where early molecular relapse detection could theoretically permit pre-emptive interventions prior to overt disease recurrence [32,33,34].

Importantly, substantial biological and methodological heterogeneity exists among circulating nucleic acid biomarkers evaluated in the transplant literature. Tumor-specific ctDNA assays, tumor-informed cfDNA-MRD approaches, total cfDNA quantification, methylation-based cfDNA profiling, cfRNA analysis, and chimerism-based monitoring are biologically distinct methodologies that should not be interpreted interchangeably. Tumor-specific ctDNA primarily reflects residual malignant clones and disease kinetics, whereas total cfDNA levels may additionally reflect tissue injury, inflammation, endothelial damage, infection, or conditioning-related toxicity. Likewise, methylation-based cfDNA and cfRNA approaches may capture immune activation, GVHD, or organ injury rather than direct molecular relapse. To address this heterogeneity, the present meta-analysis was restricted to tumor-specific ctDNA and tumor-informed/tumor-associated cfDNA studies with extractable hazard ratios, whereas non-tumor-specific approaches were synthesized narratively. Nevertheless, conceptual overlap among these biomarker categories remains an important limitation when interpreting the broader liquid biopsy literature in HCT.

Beyond relapse surveillance, several studies suggested that broader circulating nucleic acid profiling strategies may provide clinically relevant information regarding post-transplant complications. Cheng et al. demonstrated that methylation-based cfDNA profiling using whole-genome bisulfite sequencing could simultaneously inform GVHD, infection, relapse, and graft failure [23]. Similarly, highly sensitive chimerism monitoring enabled early identification of relapse risk in pediatric leukemia populations [26]. These findings suggest that future post-HCT surveillance strategies may ultimately integrate tumor-specific ctDNA with immune- and graft-related biomarkers to achieve more comprehensive multidimensional monitoring of transplant recipients [35].

Although statistical heterogeneity across pooled analyses was low, these findings should be interpreted cautiously. Only a limited number of studies contributed to each pooled endpoint, thereby reducing the statistical power of heterogeneity metrics such as the I^2^ statistic to detect true between-study variability. Moreover, substantial clinical heterogeneity remained across studies, including differences in underlying malignancies, transplant modalities, assay platforms, ctDNA targets, sampling schedules, and definitions of molecular positivity. Accordingly, the apparent consistency of pooled estimates does not necessarily imply methodological uniformity, and the magnitude of pooled hazard ratios should be interpreted within the context of relatively small observational datasets.

Another major challenge limiting broader clinical implementation is the absence of assay standardization. Included studies used heterogeneous technologies, including targeted NGS, ddPCR, patient-informed assays, multiplex PCR-NGS, methylation-based profiling, and total cfDNA quantification. Considerable variability also existed in plasma processing protocols, sequencing depth, ctDNA thresholds, timing of sample collection, and definitions of ctDNA positivity. These differences complicate cross-study comparisons and currently preclude the establishment of universally accepted thresholds for clinical decision-making. International collaborative efforts aimed at harmonizing ctDNA collection, processing, analysis, and reporting standards will therefore be essential for future clinical translation.

The potential clinical implications of ctDNA-guided monitoring after HCT are considerable. Earlier molecular relapse detection could support risk-adapted interventions such as tapering of immunosuppression, donor lymphocyte infusion, targeted therapies, bispecific antibodies, or cellular immunotherapies administered at lower disease burden [6,7,8,9,10,32]. In lymphoma and multiple myeloma, ctDNA-guided surveillance may also help identify patients who could benefit from earlier intervention prior to overt clinical relapse. However, despite these promising observations, the current evidence base remains predominantly retrospective, single-center, and observational. Consequently, ctDNA should presently be considered an emerging investigational biomarker rather than a fully established standard-of-care monitoring tool. Prospective interventional studies demonstrating improved clinical outcomes through ctDNA-guided therapeutic strategies remain critically needed.

Several limitations of this review should be acknowledged. First, only a limited number of studies contributed to each pooled endpoint, reducing the precision of pooled estimates and limiting the reliability of heterogeneity assessments. Second, most included studies were observational cohorts conducted at single institutions, increasing susceptibility to selection bias and residual confounding. Third, substantial methodological variability existed in assay platforms, timing of sampling, ctDNA targets, and endpoint definitions. Fourth, despite separating tumor-specific ctDNA studies from total cfDNA, methylation-based profiling, and chimerism-based approaches in the revised analyses, conceptual overlap among circulating nucleic acid biomarkers remains a source of complexity within the literature. Finally, although the review protocol was prospectively registered, publication bias could not be robustly assessed because relatively few studies contributed to several pooled analyses.

Despite these limitations, this study provides one of the first focused quantitative syntheses specifically evaluating tumor-specific ctDNA monitoring following HCT across multiple hematologic malignancies. The consistent associations observed between ctDNA positivity and adverse oncologic outcomes support continued investigation of ctDNA as a clinically relevant prognostic biomarker in the transplant setting. Future priorities should include multicenter prospective validation studies, assay harmonization initiatives, standardized sampling strategies, and randomized ctDNA-guided interventional trials to determine whether earlier molecular relapse detection can ultimately improve patient outcomes after HCT.

## 5. Conclusions

In conclusion, tumor-specific ctDNA positivity is consistently associated with increased relapse risk and inferior survival outcomes following hematopoietic cell transplantation. These findings support the potential role of ctDNA-based MRD monitoring as a promising non-invasive biomarker for post-transplant risk stratification and molecular surveillance. However, further assay standardization, prospective multicenter validation, and ctDNA-guided interventional trials are required before routine clinical implementation can be recommended.

## Figures and Tables

**Table 1:** Main characteristics of the studies analyzed (Clinical aspects).

Author Year Journal Country	Study Design	Patient -Count -Age -Gender	Underlying Disease/HCT Procedure	Main Findings
**ctDNA**				
Binod Dhakal et al. [20] 2022 Frontiers in Oncology USA	Retrospective cohort study	28 patients 67 years (41–70) 57.1% male	MM Autologous HCT	ctDNA positivity at 3 months post-ASCT in MM strongly predicted relapse and shorter PFS, outperforming MFC.
Sousuke Nakamura et al. [25] 2019 Blood Japan	Retrospective cohort study	53 enrolled, 51 analyzed for ctDNA Median 53 years (range: 17–68) 29 males (56.9%)	AML MDS Allo-HCT	ctDNA at 1 and 3 months post-alloSCT for AML/MDS predicted relapse and OS; increasing levels (kinetics) were a sensitive predictor.
Juhyung Kim et al. [28] 2024 In Vivo Republic of Korea	Prospective pilot study	10 patients 50–60 years 8 males	DLBCL Autologous HCT	ctDNA monitoring identified high-risk DLBCL patients who may benefit from upfront ASCT, especially with emergent poor-prognosis mutations.
Alex F. Herrera et al. [21] 2016 British Journal of Haematology USA	Retrospective cohort study	139 patients (68 for ctDNA) 59 years (27–69) 43 male (63.2%)	B-cell NHL T-cell NHL HL CLL Allo-HCT	ctDNA detection post-allo-HSCT in lymphoma predicted relapse, with positivity linked to increased progression risk and inferior PFS.
**cfDNA**				
Vanisha Patel et al. [22] 2025 Transplantation and Cellular Therapy USA	Single-center retrospective cohort study	90 patients Median 56 years (range 22–77) 49 males (54.4%)	AML allo-HCT	Detection of TA-cfDNA post-allo-HCT in AML was strongly associated with increased relapse and mortality.
Zeynep Arzu Yegin et al. [27] 2020 Balkan Medical Journal Turkey	Retrospective cohort study	177 patients 36 years (16–66) 111 male	AML ALL Mixed-phenotype acute leukemia Blastic plasmacytoid dendritic cell neoplasm Allo-HCT	Low pre-transplant cfDNA levels were associated with higher transplant-related complications and relapse in AML.
Alexandre Pellan Cheng et al. [23] 2022 Proceedings of the National Academy of Sciences of the United States of America (PNAS) USA	Prospective cohort study	27 patients Age: Not specified Gender: Not specified	Malignant hematologic disorders (n = 25) and non-malignant blood disorders (n = 2) Allo-HCT	Low-coverage bisulfite sequencing of cfDNA could simultaneously inform multiple post-HCT complications (GVHD, infection, relapse).
Sergiu Pasca et al. [24] 2023 Blood Advances USA	Prospective cohort study	82 patients median 45 years (range 33–50) 46.3% male	AML MDS MDS/MPN overlap syndrome Allo-HCT	cfDNA-MRD positivity at day 90 post-alloHCT in myeloid malignancies was strongly associated with increased relapse and inferior survival.
Miguel Waterhouse et al. [29] 2022 Cancers Germany	Prospective observational cohort study	62 patients 57 years (21–76) 37 males	AML, MDS, MPN, CMML, Aplastic Anemia Allo-HCT	Longitudinal cfDNA monitoring for MRD and chimerism enabled early relapse detection post-allo-HSCT, including extramedullary relapse.
**Chimerism**				
Anna Karen Haugaard et al. [26] 2019 Pediatric Transplantation Denmark	Retrospective cohort study	56 children (61 transplants included) Median 8.5 years (range 0.6–17.9) 41 male (67%)	ALL AML Allo-HCT	Highly sensitive RQ-PCR chimerism in pediatric ALL/AML predicted relapse ~7 months before morphological relapse, enabling early intervention

**Abbreviations:** ctDNA: circulating tumour DNA, AML: Acute Myeloid Leukemia, ALL: Acute Lymphoblastic Leukemia, GVHD: graft-versus-host disease; NHL: Non-Hodgkin lymphoma; HL: Hodgkin lymphoma; DLBCL: Diffuse large B cells lymphoma; OS: overall survival, PFS: progression free survival, MM: Multiple myeloma, HCT: Hematopoietic cell transplantation, allo-HCT: Allogeneic hematopoietic cell transplantation, MDS: Myelodysplastic Syndrome, CLL: Chronic lymphocytic leukemia, MPN: Myeloproliferative Neoplasia, CMML: Chronic Myelomonocytic Leukemia, MRD: Minimal Residual Disease, TA-cfDNA: tumor-associated cell-free DNA, MFC: multiparameter flow cytometry, RQ-PCR: real-time quantitative polymerase chain reaction.

**Table 2:** Summary of Survival Outcomes Associated with ctDNA/cfDNA Positivity.

Author (Year)	HCT Type	Disease	Outcome Measured	*p*-Value	Key Findings
**ctDNA**					
Dhakal et al. (2022) [20]	Autologous HCT	MM	PFS	0.0003	ctDNA+ at 3 months post-ASCT independently predicted shorter PFS
Nakamura et al. (2019) [25]	Allo-HCT	AML, MDS	OS	<0.01	ctDNA at 3 months predicted OS
Herrera et al. (2016) [21]	Allo-HCT	Lymphoma	PFS	0.003	ctDNA+ linked to inferior PFS
**cfDNA**					
Pasca et al. (2023) [24]	Allo-HCT	Myeloid malignancies	OS/RFS	<0.001	Day 90 cfDNA-MRD+ is strongly associated with worse OS/RFS
Patel et al. (2025) [22]	Allo-HCT	AML	OS/RFS	<0.0001	TA-cfDNA+ is associated with increased mortality
Yegin et al. (2020) [27]	Allo-HCT	AML, ALL, others	OS	0.821	No significant association
Waterhouse et al. (2022) [29]	Allo-HCT	AML, MDS, MPN, others	OS	<0.05	Longitudinal cfDNA enabled early relapse detection, impacting survival
**Chimerism**					
Haugaard et al. (2019) [26]	Allo-HCT	ALL, AML	OS	<0.05	Chimerism-based MRD predicted OS in pediatric patients

**Abbreviations:** AML = acute myeloid leukemia, cfDNA-MRD = cell-free DNA minimal residual disease; TA-cfDNA = tumor-associated cell-free DNA, OS = overall survival, PFS = progression-free survival, RFS = relapse-free survival.

**Table 3:** Summary of Studies Evaluating ctDNA/cfDNA for engraftment and Transplant-Related Mortality.

Author (Year)	Timing of Assessment	Biomarker	Outcome	Association with ctDNA/cfDNA	Notes
Yegin et al. (2020) [27]	Pre-transplant	Total cfDNA (spectrophotometry)	Transplant-related complications, mortality	Inverse: Low cfDNA is associated with higher complications/mortality	Measured total cfDNA, not tumor-specific; may reflect a different biological phenomenon
Haugaard et al. (2019) [26]	Post-transplant	RQ-PCR chimerism	Relapse, TRM	Positive: Detectable MRD associated with TRM	Pediatric population; RQ-PCR used for chimerism-based MRD
Herrera et al. (2016) [21]	Post-transplant	Targeted NGS (ctDNA)	NRM	Positive: ctDNA+ is associated with increased NRM	Lymphoma patients post-alloHCT

**Abbreviations:** HCT: hematopoietic cell transplantation; NGS = new generation sequencing, NRM = non-relapse mortality, post-alloHCT: after allogeneic HCT.

**Table 4:** Summary of Studies Reporting Association Between ctDNA/cfDNA and Relapse.

Author (Year)	HCT Type	Disease	Sample Size	ctDNA Assay	Timing of Assessment	Association with Relapse?	Key Finding
**ctDNA**							
Dhakal et al. (2022) [20]	Autologous HCT	MM	28	ddPCR (tumor-informed)	3 months post-ASCT	**Yes**	ctDNA positivity strongly predicted relapse, outperforming MFC
Nakamura et al. (2019) [25]	Allo-HCT	AML, MDS	51	Targeted NGS (patient-specific)	1 and 3 months post-alloHCT	**Yes**	ctDNA levels predictive of relapse; increasing kinetics offered high sensitivity
Herrera et al. (2016) [21]	Allo-HCT	Lymphoma (NHL, HL, CLL)	68	Targeted NGS (panel-based)	Post-alloHCT (median day +100)	**Yes**	ctDNA detection linked to significantly higher risk of disease progression
Kim et al. (2024) [28]	Autologous HCT	DLBCL	10	Targeted NGS	Pre-ASCT and post-ASCT	**Yes**	ctDNA monitoring identified high-risk patients who may benefit from upfront ASCT
**cfDNA**							
Pasca et al. (2023) [24]	Allo-HCT	Myeloid malignancies (AML, MDS, MDS/MPN)	82	cfDNA-MRD (targeted NGS)	Day 90 post-alloHCT	**Yes**	cfDNA-MRD positivity was associated with 76% 2-year CIR vs. 21% in negative patients
Patel et al. (2025) [22]	Allo-HCT	AML	90	TA-cfDNA (targeted NGS)	Post-alloHCT	**Yes**	TA-cfDNA positivity is strongly associated with increased cumulative incidence of relapse
Yegin et al. (2020) [27]	Allo-HCT	AML, ALL, mixed-phenotype acute leukemia, others	177	Total cfDNA (spectrophotometry)	Pre-transplant	**Yes**	Lower cfDNA is significantly associated with higher relapse risk in AML
Cheng et al. (2022) [23]	Allo-HCT	Malignant hematologic disorders (n = 25)	27	Low-coverage bisulfite sequencing (cfDNA)	Serial post-HCT	**Yes**	cfDNA profiling informed relapse among other complications; not the primary focus
Waterhouse et al. (2022) [29]	Allo-HCT	AML, MDS, MPN, CMML, aplastic anemia	62	Targeted NGS (patient-specific)	Longitudinal (serial)	**Yes**	Longitudinal ctDNA monitoring enabled early relapse detection, including extramedullary relapse
**Chimerism**							
Haugaard et al. (2019) [26]	Allo-HCT	ALL, AML	56 (61 transplants)	RQ-PCR chimerism	Post-transplant (serial)	**Yes**	Chimerism-based MRD predicted relapse ~7 months before morphological evidence

**Abbreviations:** HCT, hematopoietic cell transplantation; Allo-HCT, allogeneic hematopoietic cell transplantation; AML, acute myeloid leukemia; MDS, myelodysplastic syndrome; MM, multiple myeloma; ALL, acute lymphoblastic leukemia; NHL, non-Hodgkin lymphoma; HL, Hodgkin lymphoma; CLL, chronic lymphocytic leukemia; DLBCL, diffuse large B-cell lymphoma; MPN, myeloproliferative neoplasm; CMML, chronic myelomonocytic leukemia; TA-cfDNA, tumor-associated cell-free DNA; NGS, next-generation sequencing; ddPCR, droplet digital polymerase chain reaction; RQ-PCR, real-time quantitative polymerase chain reaction; cfDNA-MRD, cell-free DNA minimal residual disease; cfRNA, cell-free RNA; MFC, multiparameter flow cytometry; CIR, cumulative incidence of relapse.

**Table 5:** Risk of Bias Assessment Using the QUIPS Tool.

Study	Study Participation	Study Attrition	Prognostic Factor Measurement	Outcome Measurement	Study Confounding	Statistical Analysis & Reporting	Overall Risk
Dhakal et al. (2022) [20]	Low	Low	Moderate	Low	Moderate	Low	Moderate
Patel et al. (2025) [22]	Low	Low	Moderate	Low	Moderate	Low	Moderate
Nakamura et al. (2019) [25]	Low	Moderate	Moderate	Low	Moderate	Low	Moderate
Kim et al. (2024) [28]	Low	Low	Moderate	Low	Low	Low	Low
Herrera et al. (2016) [21]	Moderate	High	High	Low	High	Low	High
Haugaard et al. (2019) [26]	Low	Moderate	Moderate	Low	Moderate	Low	Moderate
Yegin et al. (2020) [27]	High	High	High	Low	Moderate	Low	High
Cheng et al. (2022) [23]	Low	Low	Moderate	Low	Low	Low	Low
Pasca et al. (2023) [24]	Low	Low	Moderate	Low	Moderate	Low	Moderate
Waterhouse et al. (2022) [29]	Low	Low	Moderate	Low	Moderate	Low	Moderate

**Table 6:** Studies Included in Quantitative Meta-analysis and Characteristics of Pooled Effect Estimates.

Study	Biomarker Type	Disease	Outcome Included in Meta-analysis	HR Source	Adjusted Analysis	Main Covariates Included	Included in Quantitative Synthesis	Notes
Dhakal et al. (2022) [20]	Tumor-informed ctDNA (ddPCR/mPCR-NGS)	Multiple myeloma	PFS	Directly reported HR	Yes	Age, FISH risk, MFC MRD	Yes	Post-ASCT ctDNA positivity independently predicted relapse
Nakamura et al. (2019) [25]	Tumor-specific ctDNA (targeted NGS/ddPCR)	AML/MDS	OS, relapse	Directly reported HR	No	Univariable only	Yes	Serial kinetics highly predictive of relapse
Herrera et al. (2016) [21]	ctDNA (targeted NGS)	Lymphoma	PFS	Directly reported HR	Partial	Limited adjustment	Yes	Post-alloHCT ctDNA associated with inferior PFS
Patel et al. (2025) [22]	Tumor-associated cfDNA (targeted NGS)	AML	OS, RFS, relapse	Directly reported HR	Yes	Age, donor type, CMV, conditioning, GVHD prophylaxis	Yes	Strong association with relapse and mortality
Pasca et al. (2023) [24]	Tumor-informed cfDNA-MRD (error-corrected NGS)	AML/MDS/MPN	OS, RFS, relapse	Directly reported HR	Yes	Clinical and transplant variables	Yes	Day +90 positivity is strongly associated with relapse
Waterhouse et al. (2022) [29]	Patient-specific cfDNA (targeted NGS)	AML/MDS/MPN/CMML	OS, relapse	HR was partially reconstructed from KM curves	No	Not fully adjusted	Yes	Longitudinal monitoring detected relapse early
Kim et al. (2024) [28]	ctDNA (targeted NGS/CAPP-seq)	DLBCL	Relapse	Descriptive only	No	None	No	Pilot study with insufficient HR data
Cheng et al. (2022) [23]	Methylation/WGBS cfDNA profiling	Mixed malignant/non-malignant disorders	GVHD/infection/relapse	No HR available	No	N/A	No	Included only in qualitative synthesis
Haugaard et al. (2019) [26]	RQ-PCR chimerism MRD	Pediatric ALL/AML	Relapse/TRM	Directly reported HR	No	Univariable only	No	Chimerism-based MRD analyzed narratively
Yegin et al. (2020) [27]	Total cfDNA quantity (spectrophotometry)	Acute leukemias	OS/complications	No extractable HR	Limited	Partial adjustment	No	Total cfDNA not considered tumor-specific

**Abbreviations:** ctDNA = circulating tumor DNA; cfDNA = cell-free DNA; MRD = measurable residual disease; HR = hazard ratio; OS = overall survival; PFS = progression-free survival; RFS = relapse-free survival; AML = acute myeloid leukemia; MDS = myelodysplastic syndrome; MPN = myeloproliferative neoplasm; CMML = chronic myelomonocytic leukemia; DLBCL = diffuse large B-cell lymphoma; GVHD = graft-versus-host disease; KM = Kaplan–Meier.

## Data Availability

All data generated or analyzed during this study are included in this published article.

## Abbreviations

The following abbreviations are used in this manuscript:

Disease and Clinical Terms	
ALL	Acute lymphoblastic leukemia
AML	Acute myeloid leukemia
CLL	Chronic lymphocytic leukemia
CMML	Chronic myelomonocytic leukemia
DLBCL	Diffuse large B-cell lymphoma
HL	Hodgkin lymphoma
MDS	Myelodysplastic syndromes
MM	Multiple myeloma
MPN	Myeloproliferative neoplasm
NHL	non-Hodgkin lymphoma
Transplant-Related Terms	
Allo-HCT	Allogeneic hematopoietic cell transplantation
GVHD	Graft-versus-host disease
GVL	Graft-versus-leukemia
HCT	Hematopoietic cell transplantation
NRM	non-relapse mortality
TRM	Transplant-related mortality
Molecular and Laboratory Terms	
cfDNA	Cell-free DNA
cfRNA	Cell-free RNA
ctDNA	Circulating tumor DNA
ddPCR	Droplet digital polymerase chain reaction
MFC	Multiparameter flow cytometry
MRD	Minimal residual disease
NGS	Next-generation sequencing
PCR	Polymerase chain reaction
RQ-PCR	Real-time quantitative polymerase chain reaction
TA-cfDNA	Tumor-associated cell-free DNA
WGBS	Whole-genome bisulfite sequencing
Statistical and Methodological Terms	
AUC	Area under the curve
CI	Confidence interval
CIR	Cumulative incidence of relapse
HR	Hazard ratio
NR	Not reported
OS	Overall survival
PFS	Progression-free survival
PRISMA	Preferred Reporting Items for Systematic Reviews and Meta-Analyses
QUIPS	Quality in Prognosis Studies
RFS	Relapse-free survival
Other	
APC	Article processing charges
CAR-T	Chimeric antigen receptor T-cell
